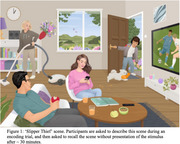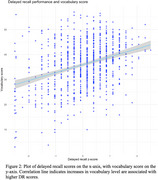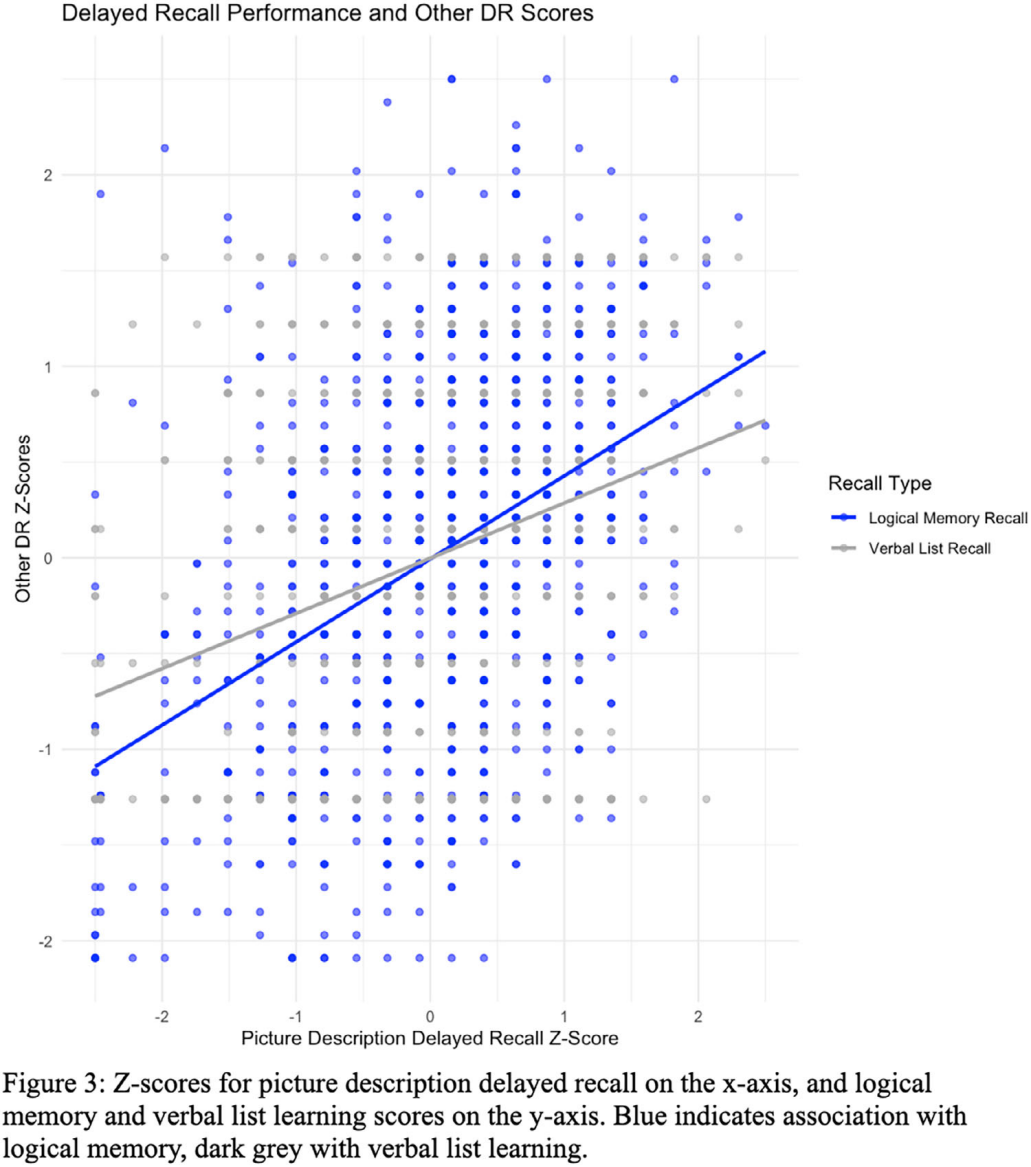# Picture Description and Delayed Recall in the California Cognitive Assessment Battery (CCAB)

**DOI:** 10.1002/alz70857_100529

**Published:** 2025-12-24

**Authors:** Kathleen Hall, Michael Blank, Kristin Geraci, Isabella Jaramillo, Omar Kahly, Miranda Miranda, Peter Pebler, David K Johnson, David L. Woods

**Affiliations:** ^1^ Neurobehavioral Systems, Inc, Berkeley, CA, USA; ^2^ UC Davis Alzheimer's Disease Center, Walnut Creek, CA, USA

## Abstract

**Background:**

Picture description (PD) tasks are widely used to evaluate speech and language ability. Here we describe the results of the traditional encoding condition as well the results of delayed picture description recall task designed to assess visuospatial memory for the picture. This task is computerized, automated, and normed for at‐home administration as part of the California Cognitive Assessment Battery (CCAB) [1].

**Methods:**

PARTICIPANTS: 772 healthy participants (55% female, 66.5 ± 8.5 years) completed the picture description and recall task in their homes during normative data collection for CCAB.

TECHNOLOGY: The “slipper thief” (Figure 1) PD task was automated with instructions delivered using text‐to‐speech. Verbal responses were digitally recorded, transcribed with consensus automatic speech recognition, and automatically scored. Participants’ performance was remotely monitored by examiners via audio and visual feeds through the CCAB's web‐browser interface.

TASK: Participants were asked to describe a visual scene (Figure 1), eliciting an average of 3.1 minutes of speech. The delayed recall trial occurred ∼30 minutes later, eliciting an average of 2.4 minutes of speech. Participants were scored based on their recall of 36 scoring elements.

**Results:**

Match counts were analyzed for PD encoding, delayed recall, and the difference between these two scores. Multiple regression analysis revealed significant effects of vocabulary (< .001), age (< .05), education (*p* < .01), and gender (< .01) on encoding and delayed recall, accounting for 23% and 14% of the variance, respectively. Delayed recall scores correlated significantly with delayed recall performance in other verbal tasks including logical memory (.42***) and verbal list learning (.28***). Forgetting, reflected in encoding‐recall difference scores and recall scores, increased with age (*p* < .001) but was not significantly influenced by other demographic factors.

**Conclusion:**

PD recall provides a novel measure of visuospatial memory for a complex scene that may assist in identifying individuals at risk for MCI and AD.

**References**

[1] Woods, D., Pebler, P., Johnson, D. K., Herron, T., Hall, K., Blank, M., … Baldo, J. (2024). The California Cognitive Assessment Battery (CCAB). Frontiers in Human Neuroscience, 17, 1305529.